# Oral Health Knowledge, Attitudes, Practices, and Literacy of Pregnant Women: A Scoping Review

**DOI:** 10.3290/j.ohpd.b4100965

**Published:** 2023-05-17

**Authors:** Annabelle Tenenbaum, Sylvie Azogui-Levy

**Affiliations:** a Dentist, Lecturer, Hospital Practioner, Department of Dental Public Health, Faculty of Dentistry, University Paris Cité; Education and Health Promotion Laboratory (LEPS) (UR 3412), UFR SMBH, University Paris Sorbonne Nord, Bobigny; AP-HP. Groupe Hospitalier Pitié Salpêtrière, Department of Oral and Dental Medicine, Paris, France. Idea and hypothesis, experimental design, wrote the manuscript.; b Dentist, Professor, Hospital Practioner, Department of Dental Public Health, Faculty of Dentistry, University Paris Cité; Education and Health Promotion Laboratory (LEPS) (UR 3412), UFR SMBH, University Paris Sorbonne Nord, Bobigny; AP-HP. Groupe Hospitalier Pitié Salpêtrière, Department of Oral and Dental Medicine, Paris, France. Idea and hypothesis, experimental design, wrote the manuscript, proofread the manuscript.

**Keywords:** health literacy, knowledge-attitude-pratice, oral health, pregnant women and child, scoping review

## Abstract

**Purpose:**

Pregnancy is a state particularly sensitive to oral pathologies (periodontal and decay). The oral health status of pregnant women can have an impact on the outcome of the pregnancy and the oral health of the child to come. As in the general population, the oral health of pregnant women is socially determined and dependent on psychosocial factors, including factors related to health behaviours. Research into the determinants of oral health in pregnant women will allow a better understanding of the mechanisms of action specific to this period of perinatality.

**Materials and Methods:**

The methodology of a scoping review was selected with the objective of investigating the contribution of knowledge, attitudes, practices (KAP) and oral health literacy on pregnant women’s oral health.

**Results:**

Of the 67 articles selected, 52 studied the ‘knowledge’ component, 27 the ‘attitude’ (including the perception and beliefs concerning health), and 54 the ‘practice’ component, while 6 articles examined literacy. The KAP components were studied in relation to socioeconomic determinants, oral health status, healthcare utilisation and oral health literacy. The level of oral health literacy of pregnant women is strongly related to their living environment and socioprofessional level which influences their attitudes and practices. Woman’s oral health practices before pregnancy can be a predictor of her practices during pregnancy.

**Conclusion:**

The complex nature of the attitude component (locus of control, sense of self-efficacy, perceived importance) is little discussed. The heterogeneity and exhaustiveness of topics related to KAP raises the question of how to more accurately assess KAP in pregnant women in a valid, reproducible, and transferable manner and the need to build a structured oral health consensus body of work. This review is a first step towards identifying the psychosocial factors that are essential for developing a model of educational intervention in oral health that combines the process of behavioural change and decision making while taking into account the concept of empowerment, and with the aim of reducing social inequalities in health.

The oral health of pregnant women is the subject of much research,^50,107^ as oral pathologies (periodontal and caries) are more frequent during pregnancy.^16,103,135^ Pregnant women have a 50% higher risk of developing gingivitis than does the general population^45,37,128^ and 51% of pregnant women have caries.^73,137^ If left untreated, these pathologies can lead to pain, infection, anxiety, and masticatory discomfort, and can negatively affect the quality of life of the pregnant woman.^29,141^ The resulting pain can also lead to the risk of self-medication and inappropriate use of analgesic drugs, which is potentially dangerous for the health of the foetus.^83,86^

It is now established that the presence of progressive periodontal disease is associated with adverse pregnancy outcomes:^147,148^ a significantly higher risk of preterm delivery^1,25,26,66,117,119,149^ and a higher incidence of chorioamnionitis (due to the effect of inflammatory mediators).^97^ There is a small but significant and independent association between periodontal disease and preterm pre-eclampsia^88^ as well as the risk of restricted intrauterine growth.^57,59^

Furthermore, microorganisms (including bacteria that initiate the caries process) can be found very early in the oral cavity of newborns due to bacterial transmission from the oral cavity of the mother to the child.^61,140^ Thus, the earlier the oral bacterial colonisation from mother to child occurs, the higher is the risk of caries development in the child. The results of the Dunedin (New Zealand) cohort showed that having more than one missing tooth at 32 years of age and a high caries rate leads to a higher risk of caries in the child (after controlling for social and oral hygiene levels).^120^

Models have been developed to better understand the determinants of oral health in children. Fisher-Owens presented a conceptual model with three dimensions (individual, family, and community) through a biopsychosocial approach to the caries process.^39^ In 2012, Quissel et al proposed a model detailing the individual level using sociodemographic factors, expected mediators, possible moderators, and health behaviours acting as a chain of determinants leading to oral health events.^105^ Expected mediators are oral health knowledge and attitudes, possible moderators are psychological influences and other risk factors, including health behaviours (adherence to oral health recommendations, use of the healthcare system) and health literacy. According to Sørensen et al,^125^ health literacy involves “an individual’s knowledge, skills, motivation and ability to identify, understand, evaluate and use health information when making decisions in the contexts of health care, disease prevention and health promotion to maintain or improve the quality of life over the life course”. Three levels of measurement have been defined. The functional level refers to the reading and writing skills needed to cope with everyday situations. The interactive level includes more advanced skills that allow the patient to be active in information seeking and communication. Finally, the critical level corresponds to even more advanced skills, allowing critical analysis of information and greater control over one’s health.^93^ However, a growing number of studies have shown that not only a significant proportion of the population has difficulty mobilising these skills and does not have access to the information and exchanges that enable them to be autonomous in matters of health,^6^ but also that people with low health literacy are less likely to adopt health-promoting behaviours, participate in screening programmes, and use preventive services.^91,95,96,134^ Oral health literacy (OHL) has been shown to be associated with oral health status.^32,76,62^ In the USA, a study by Lee et al^72^ found an improvement in self-reported oral health status with increasing levels of OHL and feelings of self-efficacy.

For pregnant women, low OHL can have a negative impact not only on their own oral health and pregnancy outcome but also on the oral health and preventive behaviours of the child.^5,19,82,122,126^ Most studies show that OHL is influenced by social or psychosocial determinants and related to the level of oral health knowledge. Pregnant women with a low level of OHL are less likely to have favourable oral health behaviours and will have children with less favourable oral health behaviours (inadequate toothbrushing, sweetened foods).

Oral health status indicators also depend on social and environmental determinants, in addition to the use of healthcare (linked to the healthcare system).^108,143^ The existence of disparities between income, social level, and oral pathologies has been shown for each age group and reflects social inequalities in health.^74^ Thus, there is a social gradient in relation to the prevalence or incidence of oral pathologies.^4,69,71,79^ Socioeconomically disadvantaged people are more likely to have untreated oral diseases and unmet oral care needs than those at the top of the social ladder.^28,116,138^ This is also true for the pregnant population; women who are more likely to have a prenatal dental check-up belong to the middle and upper-middle classes, have private health insurance, live in urban areas, and have a higher level of education.^77,78,111,124^ Studies show a correlation between the prevalence of parental caries and parental characteristics during pregnancy (e.g. mother’s age, family status, income, education level, and country of origin) and the prevalence of children’s caries at five years of age.^90,145^ Hence, there are social disparities in the use of dental care.

Research into the determinants of oral health of pregnant women would allow a better understanding of the mechanisms of action specific to this period of perinatal life. This research is necessary for the implementation of oral health promotion interventions and their adaptation to meet the needs of pregnant women. Educational interventions for pregnant women during pregnancy have proven to be beneficial in terms of knowledge transfer and are a unique opportunity to develop skills, confidence concerning parenthood, and self-esteem.^92^ Educational interventions to promote good oral health in prenatal care must take the knowledge, attitudes, and practices, as well as OHL, of pregnant women into account. The aim of this study was to analyse these mechanisms through a scoping review of pregnant women’s knowledge, attitudes, and practices concerning oral health and their OHL.

## Materials and Methods

**Fig 1:**
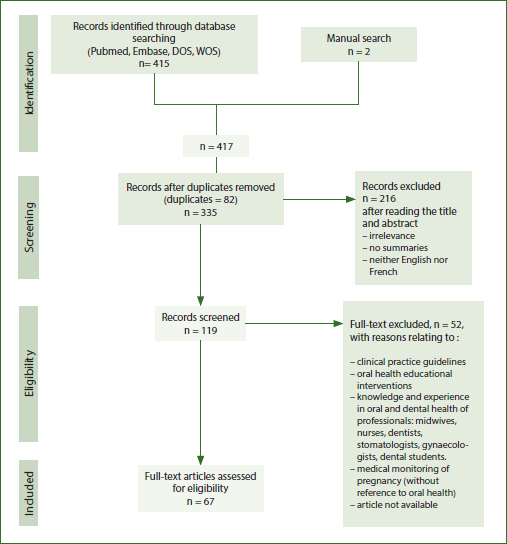
PRISMA flow chart.

A scoping review was chosen as the research methodology because it enables rapid exploration of key concepts, a review of the available literature, identification of the types of evidence, and identification of knowledge gaps. The primary method is appropriate for the exploration of our topic, which has not yet been studied using the methodology applied in this study, and for which the extent of research that could be associated with it is unknown. The Johanna Briggs Institute methodological framework and recommendations and the PRISMA-ScR checklist (Fig 1) were implemented to conduct this scoping review.^101,104^

A systematic literature search was conducted using the four electronic medical and dental databases MEDLINE (via PubMed), Embase / Ovid, Dentistry and Oral Sciences Sources (DOS), and Web Of Science (WOS) between January and February 2023.

The main search terms, in MeSH, free text, or keyword format using the search algorithm via PubMed were: (((((((pregnant OR pregnancy)) OR “Pregnancy”[Mesh])) AND ((“Oral Health”[Mesh]) OR ((“dental health” OR “oral health”)))) AND ((((“oral health literacy” OR “health literacy” OR “literacy” OR “oral health knowledge” OR “health knowledge, attitudes, practice”[mh] OR “oral health behavior” OR “oral health behaviors” OR “oral health behaviour” OR “oral health behaviours” OR “health belief model” OR “health belief”))) OR “Health Literacy”[Mesh]))) NOT (intervention*[ti] OR session*[ti] OR education*[ti] OR program[ti] OR message*[ti] OR training[ti]).

The ‘age’ filter was not activated and no date limit was set. An additional hand search completed the selection of studies.

Articles were first selected on the basis of their titles; then the abstracts were independently reviewed by the authors of the current study. The inclusion and exclusion criteria were discussed in detail to ensure agreement between the two authors in the selection of studies. Both authors then independently reviewed all titles and abstracts based on these criteria. The exclusion criteria were languages other than French or English, lack of an abstract, and content not relevant to the study objective. The inclusion criteria were articles in English or French, available abstracts, and relevant content dealing with the knowledge, attitudes, practices, and literacy of pregnant women in relation to oral health. Eligible articles were read in full.

The included articles were then grouped according to the components investigated in the study. Data extracted for all studies included: name of the first author, year, country, methodology used, number of participants in the study, study site, data collection tools and questionnaire administration (self-administered, guided, interview), purpose, and variables (KAP and literacy).

## Results

The search strategy identified 417 articles. After removing duplicates, 335 potential articles remained. After reading the title and abstract, 216 articles were excluded. In total, 119 studies were selected for full-text reading. Forty studies were subsequently excluded due to irrelevant content. Finally, the selection process resulted in the inclusion of 67 articles.

### Characteristics of the Studies Included

Among the selected articles (Table 1), the title referred to at least one of the knowledge, attitude, or practice components in 42 articles and to literacy in six papers.

**Table 1:** Summary of selected studies

Author, year, place of study [Reference]	Study design (frame, duration)	Sample size	Objectives	Data collection tools	Administration	DCC*	SDD*	LSO*	KAP*	Main results
Abiola et al, 2011 Nigeria [2]	Cross-sectional study Unicentric 6 months	453	Describe oral hygiene knowledge, attitudes, and habits	Questionnaire designed by the author	Self-administered		×		×	The relationship between oral health knowledge and ethnicity, education level, and pregnancy trimester were statistically significant. Women’s knowledge and attitudes towards oral health were not reflected in their oral hygiene practices.
Alwaeli et al, 2005 Jordan [7]	Cross-sectional study Multicentric 4 months	275	Assess the level of knowledge and awareness of periodontal health	Questionnaire designed by the author	Self-administered		×		×	A minority of pregnant women had the knowledge or ability to identify plaque. 2/3 of the pregnant women knew the main cause of gingivitis and half did not think it was necessary to increase the frequency of brushing their teeth during pregnancy. Only 5.1% thought there might be a link between periodontal disease and preterm delivery
Amin et al, 2014 Canada [8]	Cross-sectional study Unicentric 5 months	423	Identify and assess factors affecting the use of dental services during pregnancy	Questionnaire designed by the author	Self-administered		×		×	19.2% of mothers reported difficulties brushing their teeth and 25% had dental/periodontal problems. Half of the participants had a dental visit during their pregnancy. Canadian-born women were 48% more likely to visit a dentist during pregnancy than their non-Canadian counterparts. Education, dental insurance, and household income were also positively associated with usage patterns. Mothers who were more aware of the possible links between oral health and pregnancy and those who visited the dentist every six months were more likely to visit the dentist during pregnancy.
Avula et al, 2013 India [9]	Cross-sectional study Multicentric 4 weeks	359	Assess the knowledge, attitudes, and practices of pregnant women regarding oral hygiene and their potential relationship to pregnancy outcomes, identify the various risk indicators for gingival bleeding in pregnancy.	Questionnaire designed by the author	Administered face-to-face		×		×	Most women were unaware of the importance of oral hygiene and its likely association with adverse pregnancy outcomes. The risk indicators identified for gingival bleeding during pregnancy were poor knowledge of the various parameters, brushing teeth only once a day, and infrequent visits to the dentist
Bahramian et al, 2018 Iran [10]	Qualitative research multicentric 2 weeks	22 pregnant women 8 midwives 12 dentists	Explore the barriers and factors that influence pregnant women’s use of dental services.	Interview guide designed by the author	Semi-structured face-to-face interview group discussion		×		×	Factors identified as barriers to utilization include lack of knowledge, cost of dental care, physiological changes, fear and other psychological conditions, time constraints, reluctance of dentists to accept the treatment of pregnant women, cultural taboos, and lack of interprofessional collaboration
Baker et al, 2016 United States [11]	Secondary analysis of data collected in a cross-sectional study case - retrospective control Unicentric Period not specified	268 / 186 (454)	Examine the influence of being a mother on pregnant women’s knowledge and beliefs about children’s oral health.	Questionnaire designed by the author	Self-administered (oral if necessary)		×		×	The child oral health knowledge indicator was affected by ethnicity, maternal self-assessment of oral health, and previous pregnancies. The infant oral health belief indicator related to child oral health was influenced by maternal oral health beliefs and history of access to dental care.
Balan et al, 2018 Singapore [12]	Cross-sectional study Unicentric 7 months	82	Examine the correlations between oral health knowledge, attitudes and practices among pregnant women.	Questionnaire designed by the author based on the PRAMS (Pregnancy Risk Assessment Monitoring system), ROHKI (Rustvold Oral health knowledge inventory, and OHAQ (oral health attitudes questionnaire)	Self-administered	×	×		×	Women in the lowest income group had more dental problems reported during pregnancy than those in the highest income group. Statistically significant positive correlations were found between the scores of the attitude, practice, and oral health knowledge levels. Plaque index scores negatively correlated with oral health practice scores.
Bamanikar et al, 2013 Oman [13]	Cross-sectional study Unicentric 1 month	95	Assess women’s knowledge and attitudes towards oral health during pregnancy and examine their oral health self-management practices	Questionnaire designed by the author	Self-administered				×	Knowledge of dental care was low among pregnant women. Although most agreed that women should have a dental examination during pregnancy, only half actually did so.
Barbieri et al, 2018 Brazil [14]	Cross-sectional study unicentric 5 months	195	Assess oral health knowledge and associated sociodemographic factors of women	Questionnaire adapted from Frazao	- Self-administered - group meeting				×	Education of 8 years or more and having one or two children were associated with adequate oral health knowledge.
Bhaskar et al, 2020 India [15]	Cross-sectional study unicentric period not specified	400	Assesses self‐perception of oral health knowledge and related behaviours among antenatal mother	Questionnaire designed by the author	Not specified				×	- Poor oral health knowledge was observed among 3/4 of the pregnant mothers. - Oral health problems were reported by 63.2% of them. - Majors reasons for delaying dental services: low priority for oral health, fear for fetal safety - A better oral health knowledge was observed among the upper middle class, who had visited dentists within the last six months and already parents.
Boggess et al, 2010 USA [17]	Cross-sectional study unicentric 3 months	599	Examine women’s oral hygiene practices and use of dental services during pregnancy	Questionnaire designed by the author	Self-administered Group meeting				×	- Being over 36 years of age, being Hispanic, having an annual income of less than $30,000, not flossing often, and not having regular dental care in general (outside of pregnancy) were significantly associated with not having dental care during pregnancy. - A woman’s lack of routine dental care prior to pregnancy was the most significant predictor of not receiving routine dental care during pregnancy.
Boggess et al, 2011 USA [18]	Cross-sectional study unicentric 3 months	599	Assess and compare mothers’ oral health knowledge and beliefs and determine whether maternal ethnicity or other maternal factors contribute to women’s knowledge or beliefs	Questionnaire designed by the author	Self-directed				×	The oral health knowledge scores of Hispanic women were significantly lower than those of African American women. Education level of 8 years or less was significantly associated with a lower oral health belief score.
Buerlein et al, 2011 USA [21]	Qualitative research Multicentric 1 year	34	Obtain information on the knowledge, beliefs, and practices of low-income women regarding oral health during pregnancy and infant care	Semi-structured interview guide designed by the author	Focus groups				×	The women were reasonably well informed about oral health practices for themselves and their children. However, significant myths and misperceptions persist. Most women had not received oral health information in time to apply it according to recommended practices.
Chawlowska et al, 2022 Poland [23]	Cross-sectional study Multicentric Two weeks	400	Investigate interactions between oral health behaviours, knowledge, and literacy of Polish expectant mothers	Questionnaire designed by the author	Administered face-to-face				×	Knowledge and literacy scores were associated with, among other things, maternal education, selected oral hygiene practices, and reported extractions of permanent teeth. Insufficient awareness of caries as an infectious disease and of the appropriate timing for the child’s first dental visit were noticed. Self-assessment of oral health status tended to be overly optimistic and not linked to the self-reported outcomes of the pregnant women.
Chung et al, 2014 USA [24]	Cross-sectional study Multicentric	99	Describe the oral status of a sample of pregnant women, examine the relationships between socio-demographic factors, oral health, behavioural factors	Questionnaire developed from the National Health and Nutrition Survey and the Maternal and Child Health Assessment.	Administered face-to-face or by telephone				×	With respect to behaviours, lack of dental visits in the past six months was associated with poorer oral health status. Education level, ethnicity, lower income, and not having insurance were related to poorer oral health status.
Correia et al, 2017 England [27]	Prospective study Unicentric Period not specified	115	Determine the oral health knowledge of pregnant women and share their future plans for the child’s dental care	Questionnaire designed from the 2009 British Adult Dental Health Survey (ADHS).	Self-administered				×	Lack of knowledge about foods that can cause cavities. Most pregnant women planned to read or seek advice before buying their child’s first toothpaste. No difference in knowledge of prevention tools (diet and fluoride supplements) between first-time pregnant women and those who already had children.
Delemotte Valcarcel Tramini et al, 2013 France [30]	Cross-sectional study Unicentric 3.5 months	203	Assess oral and dental health according to certain socio-demographic factors in pregnant women and offer a dental examination at the same time as the prenatal interview of routine	Spices questionnaire and questions designed by the author	Oral examination and oral questionnaire at the prenatal examination					The average number of decayed teeth was significantly higher in the precarious group than in the non-precarious group. No significant difference in periodontal damage. In general, the knowledge of good dental hygiene and specific preventive measures during pregnancy attenuated the differences in oral health status related to poverty.
Dinas et al, 2007 Greece [33]	Cross-sectional study Unicentric 1 year	425	Explore the dental status and use of dental health services of pregnant women during pregnancy, as well as their perceptions of potential associations between dental care during pregnancy and pregnancy outcomes.	Questionnaire designed by the author	Self-administered					Gingivitis in pregnancy was independently associated with being of non-Greek origin, multiparity versus primiparity, lower economic class versus middle and upper class, and lack of routine primary dental care. Most women who consulted during pregnancy thought that dental treatment during pregnancy could have a negative effect on the outcome of the pregnancy. The presence of gingivitis during pregnancy and the belief that dental treatment during pregnancy is safe were both independently associated with visiting a dentist during pregnancy.
Eigbobo et al, 2013 Nigeria [34]	Cross-sectional study Unicentric 4 weeks	706	Assess the knowledge and awareness of expectant mothers of factors that influence the oral health of children.	Questionnaire designed by the author	Self-administered					A statistically significant proportion of the women had a moderate to high score in knowledge of causative and preventive factors of caries and periodontal disease, with high knowledge of the role of bacteria in oral health and the likely transmission of bacteria from mother to child, but only 1/3 of women knowing not to leave a bottle/breast in a sleeping child’s mouth. More than half of women do not know that temporary teeth are treatable. Less than 1% of mothers had seen a dentist for their children.
Fadavi et al, 2009 USA [36]	Cross-sectional study Unicentric Period not specified	111 (50 AA, 61 HA)	Compare dental visits and oral health knowledge among African American pregnant adolescents (AA) and Hispanic American (HA) women in a community health clinic	Questionnaire designed by the author, adapted from the Habashneh* questionnaire	Self-administered					Predictive factors for consulting during pregnancy: living as a couple and consulting regularly before pregnancy.
Gaffar et al, 2016 Saudi Arabia [40]	Cross-sectional study Unicentric 3 months	197	Assess the association between oral health knowledge and practices of Saudi pregnant women	Questionnaire designed by the author	Self-administered		×		×	Oral health knowledge was not significantly associated with the implementation of oral hygiene practices. Women who regularly visited a dentist were more likely to know how to prevent caries in their child, and that dental treatment during pregnancy and infant health were correlated.
Gambhir et al, 2015 India [41]	Systematic review	×	Assess oral health knowledge and awareness among pregnant women in India							
Gaszynska et al, 2015 Poland [42]	Cross-sectional study Multicentric 2 weeks to 1 month	1380	Assess the level of oral health knowledge and determine the oral health status of pregnant women in Poland	Empirical data were obtained from the national surveillance of oral health and its determinants. Dental health awareness questionnaire designed at the Medical University of Lodz (Franciszek Szatko. )	Self-administered	×	×		×	More than 60% of pregnant women rated their knowledge and practical skills in caring for their own and their unborn child’s teeth as limited, inadequate, or non-existent.
Georges et al, 2013 Australia [43]	Cross-sectional study Unicentric 20 weeks	241	Conduct a review of the oral health status, knowledge, and practices of pregnant women in south-west Sydney	Questionnaire designed by the author	Self-administered		×		×	There is a significant difference in the use of dental services among pregnant women with the following factors: higher family income, private health insurance, having received information on perinatal oral health, and knowing the status of their own oral health.
Gupta et al, 2015 India [46]	Case-retrospective control Multicentric 15 months	200/200, at random	Determine the oral health knowledge and attitudes of pregnant women Assess oral hygiene practices of pregnant women Assess their knowledge of the link between oral health and pregnancy outcomes. Compare these results with women who are not pregnant Assess whether their awareness of dental care increased after conception	Questionnaire designed by the author	Questionnaire verbally explained to illiterate women		×		×	Virtually no information is provided by the gynaecologist regarding the impact of oral health on pregnancy outcome. Only 3% of pregnant women were aware that oral health correlated with adverse pregnancy outcomes.
Gupta et al, 2019 Nepal [47]	Qualitative research Multicentric 6 months	55	To acquire data on knowledge and practices of pregnant women regarding oral health, common dental problems in pregnancy, identify the barriers in seeking care for dental problems in pregnancy	Interview guide designed by the author	Administered face-to-face		×		×	Almost all the women acknowledged that routine dental care was needed for health. Only 12% were aware that poor dental health could affect the baby’s weight. Only 10% of pregnant women had seen a dentist in between 6 to 12 months. Inadequate oral hygiene habits of 1/3of the women indicate inadequate knowledge about maternal and infant oral health, especially relating to good oral hygiene habits during the prenatal period. For half of the women oral health was not seen as priority (barriers expressed: costs of treatment and safety concerns).
Habashneh et al, 2005 USA [48]	Cross-sectional study Unicentric 7 months	625	Study factors related to the use of dental services during pregnancy and Assess the extent of mothers’ oral health knowledge and the effect on the outcomes of the pregnancy	Questionnaire designed by the author	Self-administered		×		×	Factors significantly associated with reporting dental visits during pregnancy were personal factors (being married, visiting the dentist more often outside of pregnancy, and using interdental brushes), financial factors (dental insurance), and knowledge of the possible link between oral health and pregnancy outcome.
Hom et al, 2012 USA [51]	Cross-sectional study Multicentric	119	Determine the levels and examine the associations between oral health literacy (OHL) and oral health knowledge among low-income, first-time pregnant patients	REALD-30	Face-to-face interview		×	×	×	Presence of a positive correlation between OHL and oral health knowledge.
Hosseintalaei et al, 2017 Iran [52]	Cross-sectional study Multicentric 1 year	300	To explore the association of perceived susceptibility and self-efficacy with the index of decayed, missing and filled teeth (DMFT) of pregnant women.	Questionnaire based on Shamsi et al 2012, derived from Health Belief Model	self-administered		×		×	There is a significant and inverse relationship between: the average DMFT and the average knowledge scores, the disease risk perception scores, and the perceived self-efficacy score; their level of education and missing teeth; and the average DMFT and the economic condition of pregnant women. In addition, there is a statistically significant, positive correlation between educational level and filled teeth.
Hu et al, 2022 China [53]	Cross-sectional study Unicentric 1 month	- 224	Investigated the oral health status and knowledge amongst pregnant women in Shanghai	Questionnaire designed by the author and developed from the Fourth National Oral Health Questionnaire	Self-administered	×	×		×	Oral health status among pregnant women was poor. Oral health awareness and attitudes were relatively high. Mismatch between oral health knowledge, awareness and behaviours
Hullah et al, 2008 England [54]	Cross-sectional study Unicentric 2 months	206	Describe self-reported oral health, oral hygiene habits, frequency of dental visits and factors associated with dental attendance among pregnant women in a North London hospital, the majority of whom are immigrants.	Questionnaire designed by the author	Self-administered		×		×	Dental attendance was low and the average time since their last dental visit was 1.8 to 1.61 years. More than a third of the women surveyed were not aware of free dental care during pregnancy and the following 12 months. Only 36% of the women surveyed visited a dentist regularly. Pregnancy has not changed their attitude towards dental care. There does not appear to be any difference in attitudes to dental care between pregnant immigrants and British-born women.
Hunter et al, 2011 USA [55]	Descriptive, retrospective and correlational study multicentric 23 months	380	Describe the oral health status and oral health practices of low-income pregnant women in San Diego, California Determine the oral health care education needs of this population	Medical records Questions from the OHAQ oral health assessment questionnaire	Medical records	×	×		×	- Hispanic women needed more dental care than Filipino women or women of other ethnicities. - The results suggest that low-income pregnant women have certain adequate oral health care practices but need access to dental care and oral health education.
Ibrahim et al, 2017 Sudan [56]	Cross-sectional study Unicentric 8 weeks	420	Assess the oral health status, knowledge, attitudes and practices of a sample of Sudanese pregnant women to obtain data Necessary for the implementation of a health prevention program on oral health during pregnancy	The questionnaire was adapted by the research team from standard questionnaires	Face-to-face interview	×	×		×	12% of women had good oral health knowledge and 21.2% had a positive attitude Most women had poor oral hygiene practices Only 20% had visited a dentist during their pregnancy.
Javali et al, 2022 India [58]	Cross-sectional study Multicentric Period not specified	445	Assess the knowledge, attitude, and practices of oral health among preg- nant women in South India	Questionnaire designed by the author	Self-administered		×		×	Critical gaps in knowledge (good level) and practice of oral and dental healthcare of pregnant women were observed. Pregnant women showed poor compliance with the recommended protocol: did not practice oral hygiene as recommended and were reluctant to routine dental visits during pregnancy. Myths and barriers to dental treatment during pregnancy were observed Women with lower educational levels knew less about the beneficial effects of fluoride toothpaste.
Kateeb et al, 2018a/b Palestine [63]	Cross-sectional study multicentric 8 months	151	Describe the experience of Palestinian pregnant women with dental caries and examine the relationship with their oral health knowledge, beliefs, and behaviours and their access to dental care	A structured questionnaire was developed based on previous studies MSL Instrumental Social Support	Face-to-face interview	×	×		×	It was shown that women who had graduated from high school had lower DMFT scores than women who had not. Women who had visited a dentist in the past 6 months had a higher DMFT score than women who had never visited a dentist. Women who thought they might lose a tooth simply because they were pregnant had a high DMFT score. Age, education level, recent dental visit and belief that it is not safe to obtain routine dental care during pregnancy explained 25% of the variation in the DMFT score.
Keirse et al, 2010 Australia [64]	Cross-sectional study Multicentric 2 years	649	Assess pregnant women’s opinions and perceptions of oral health and their relationship with oral hygiene and dental care practices	Self-completed questionnaire Dental anxiety care	Self-administered		×		×	The fact that oral health services are available free of charge will not help if women do not know about them, do not use them because they do not perceive a problem, do not consider them necessary, or think that dental care should be avoided during pregnancy.
Kobylinska et al, 2020 Poland [65]	Cross-sectional study Electronic survey on the Web – one website 2 months	2480	To identify and determine the effects of sociodemographic and pregnancy-related factors on oral health attitudes during pregnancy as well as the main predictors of proper oral practices	Questionnaire designed by the author	Self-administered		×		×	Correlation was found between level of knowledge, health behaviours and sociodemographic factors Proper health behaviours were more strongly correlated with the level of knowledge and the use of dental care before pregnancy rather than age, good or very good financial status, high level of education, urban residence or occupational activity 2/3 of the women reported a dental check-up during pregnancy, dental visits depended on the conviction about their safety.
Lakhani et al, 2014 Pakistan [67]	Cross-sectional study unicentric 9 weeks	118	Assess pregnant women’s attitudes towards oral health during pregnancy and examine their oral health self-care practices	Questionnaire designed by the author	Unknown	×	×		×	The study highlighted the lack of awareness of oral health maintenance.
Lakshmi et al, 2020 India [68]	Cross-sectional study Unicentric 4 months	606	Assess oral health knowledge of pregnant women visiting Government Maternity Hospital, Hyderabad.	A structured questionnaire was developed and validated on previous studies	Self-administered		×		×	Inadequate knowledge with regard to oral health was observed among half of the respondent (3/4 did not know that brushing with fluoridated toothpaste prevent caries, and that every painful tooth should not be removed). The current study identified participant’s lacuna on the interdependent relationships between pregnancy affecting oral health and vise versa poor oral health affecting pregnancy outcomes. The respondents who never visited dentists had low knowledge scores.
Lazaridi et al, 2022 Switzerland [70]	Cross-sectional survey Multicentric 17 months	385	Evaluate the knowledge and practices of Swiss women regarding oral health during pregnancy	Questionnaire designed by the author	Self-administered		×		×	The pregnant women are moderately informed about the importance of good oral health maintenance during pregnancy to diminish the risks of complications. The participation of health-care professionals is too limited.
Llena et al, 2019 Spain [73]	Cross-sectional survey Multicentric 6 months	139	Assess the knowledge of pregnant women in terms of oral health and prevention, correlating with socio-sanitary, educational factors, self-care and oral health state	A structured questionnaire was developed and validated on previous studies	Questionnaire designed by the author adapted from previous study.		×		×	Level of education, nationality, self-care, and knowledge on prevention and oral health were the factors that determined a greater level of general knowledge on oral health from the pregnant women
Lubon et al, 2018 Nepal [75]	Qualitative research Multicentric 5 months	39	Understand the dental care-seeking behaviour, oral health knowledge, and attitudes of pregnant women in rural Nepal	Interview guide designed by the author	Semi-structured in-depth interviews (SIIs) and group discussions (FGD)		×		×	The women in this community were unable to correctly identify the signs and causes of caries and periodontal disease and did not know where to find dentists. If there was no pain, most women said there was no need to see a dentist. In this population, delays in seeking care for maternal and neonatal complications were attributed to the low perception of disease severity, even when symptoms were recognised early (both for general and dental care). This study suggests that standardised, culturally appropriate and simple educational messages need to be developed and disseminated through appropriate behaviour-change–communication approaches.
Martinez-Beneyto et al, 2011 Spain [80]	Cross-sectional study Unicentric 23 months	337 questionnaire / 282 dental examination	Study the relationship between self-reported oral hygiene habits and oral status (caries and periodontal index) I of pregnant women	Questionnaire designed by the author	Self-administered	×	×		×	The present study showed that pregnant women with a positive perception of oral health had a lower DMFT score and periodontal index.
Massoni et al, 2015 Brazil [81]	Cross-sectional study Unicentric 1 month	100	Conduct a comparative assessment of primiparous and multiparous mothers’ knowledge of caries disease, access to information, and use of dental services during pregnancy	Questionnaire designed by the author	Unknown		×		×	Pregnant women generally associate oral health with general health, but do not seek dental care during pregnancy. Participants have little understanding of the multifactorial nature of this disease.
Maybury et al, 2019 USA [82]	Mixed method: cross-sectional study and qualitative research Multicentric 21 months	117	Determine the impact of oral health literacy (OHL) on use of prenatal dental care and knowledge, understanding, and practices related to preventing dental caries (tooth decay) among low-income pregnant women in Maryland.	Questionnaire designed by the author and developed from the Consumer Assessment of Healthcare Providers and Systems (communication practices), Chew et al’ brief health literacy screening tool and Maternity Social Support Scale (MSSS) by Webster et al for social support Structured interview guide developed by the author	Self-administered questionnaire, one-on-one interview or focus group		×	×	×	53% of participants reported seeing a dentist during their pregnancy. Most women were unaware of the Medicaid dental program for pregnant women and the importance of prenatal dental care. They could not afford care if there was an associated cost and had difficulty finding a Medicaid dental provider. Most study participants had adequate health literacy. However they lacked understanding of how to prevent caries and did not practice behaviours to prevent this disease, which invalidate the results of health literacy level.
Mofidi et al, 2009 USA [84]	Qualitative researc Multicentric 3 months	31 staff members, 22 parents and 13 pregnant women.	Explore the oral health knowledge, attitudes and activities of Early Head Start (EHS) staff, parents and pregnant women	Interview guide designed by the author	Guided discussion group		×		×	Gaps have been noted in the oral health activities of the EHS (Early Head Start) programs. Participants expressed confusion about the application of the oral health performance criteria. The need for culturally appropriate, hands-on oral health education was highlighted.
Moghadam et al, 2013 Iran [85]	Cross-sectional study 1 year	149	Assess the periodontal health knowledge, attitudes and practices of pregnant women in relation to the association between periodontal disease and pregnancy outcomes	Questionnaire designed by the author	Self-administered		×		×	The correlation between practice and attitude was not statistically significant, but there was a significant linear correlation between attitude and knowledge. This study found that the mean scores for knowledge, attitude, and practice increased with higher education, but their differences were not statistically significant.
Naavaal et al, 2022 USA [87]	Cross-sectional study Unicentric 8 months	187	Examine and compare pregnancy-related oral health knowledge and barriers to dental care access during pregnancy among women with private and public insurance Estimate awareness of available Medicaid pregnancy dental benefit among Medicaid-enrolled women and explore associated factors.	Questionnaire designed by the author, developed on previous study	Self-administered		×		×	There was a significant gap in dental care use and knowledge between Medicaid-enrolled and privately insured women. 1/3 of the Medicaid-enrolled women was unaware of the Medicaid pregnancy dental benefit. Medicaid-enrolled women reported a lower prevalence of a routine dental checkup in the past year, lower knowledge scores, and more barriers to accessing dental care during pregnancy compared with privately insured women. Awareness did not differ by pregnancy status.
Nagaraj et al, 2012 India [89]	Cross-sectional study, case / control Unicentric Period not specified	Group A 170 pregnant women Group B 170 mothers of children up to one year of age	Assess the knowledge, attitudes, and practices of pregnant women and mothers regarding the eating habits and oral health of children	Two separate questionnaires A / B designed by the author	Structured interview in the form of a questionnaire completed by the interviewer		×		×	A lack of knowledge about infant feeding and weaning was noted in both groups. One positive finding was that most mothers/pregnant women cleaned their child’s mouth using cotton gauze. Both groups consider primary teeth to be important in the oral cavity.
Obuna et al, 2012 Nigeria [94]	Cross-sectional study Unicentric 3 months	363	Determine the level of knowledge concerning pregnancy-related oral diseases and the level of utilization of dental services by pregnant women attending the prenatal clinic of a university hospital	Questionnaire designed by the author	Self-administered (or physician-assisted)		×		×	The study found - poor overall health behaviour of the population. - low oral health knowledge and use of dental services.
Ozen et al, 2012 Turkey [98]	Cross-sectional study Unicentric Period not specified	351	Assess women’s knowledge and behaviour regarding oral health care during pregnancy	Questionnaire adapted from Habashneh et al	Face-to-face interview		×		×	Despite the high level of education, 73% of women still believed that calcium would be extracted from their teeth by the developing baby and 43% believed the erroneous statement “One tooth for one baby”. 68.7% of women had oral health problems during their pregnancy; however, only 13.7% consulted a dentist during their pregnancy.
Penmetsa et al, 2018 India [99]	Cross-sectional study Multicentric 6 months	260 Group A 130/ B 130	Assess knowledge of oral health in pregnant women based on their dental visit	Self-validated questionnaire	Explained verbally		×		×	The study population had very low awareness of oral health and its relationship to pregnancy. In addition, most of the study population was unaware that dental treatment was safe during pregnancy.
Pentapati et al, 2013 India [100]	Cross-sectional study Multicentric 3 months	386	Assess the knowledge of caries disease among pregnant women and the relationship with sociodemographic characteristics and caries experience in rural India.	Questionnaire designed by the author adapted from Nakazano et al	Self-administered		×		×	Women with higher levels of education were more aware of the appearance of decayed teeth, the serious problems associated with them, the use of fluoride toothpaste, and the consumption of sweets. Higher levels of education can lead to greater knowledge of and access to healthcare information, as well as the ability to access healthcare services.
Petit et al, 2021 France [102]	Cross-sectional study Multicentric 3 months	212	Evaluate periodontal knowledge and behaviour related to the oral health of pregnant women and to determine influencing factors during pregnancy follow-up in a French population	Auestionnaire designed by the author	Self-administered		×		×	92% of the women considered prevention of oral disease during pregnancy important. Information related to oral health was given by pregnancy professionals to only 18.2% of the sample population. Women aware of possible dental consultation during pregnancy and with a higher level of knowledge were more prone to have a high self-perceived oral health status, regularly consult their dentist and have visited an obstetrician/gynaecologist. Women considering prevention and management of dental diseases during pregnancy of importance have been seen during pregnancy by their dentist. (having visit a dentist during the year before is a factor influencing visiting during pregnancy)
Rahbari et al, 2015 USA [106]	Cross-sectional study Multicentric Period not specified	103 (56 pregnant women and 47 mothers)	Assess the oral health knowledge and behaviours of pregnant women and mothers of young children in relation to early childhood caries to evaluate the need for an oral health education program	Questionnaire designed by the authors	Self-administered		×		×	There was a statistically significant correlation between mothers’ ratings of their oral health and frequency of brushing + flossing and between mothers’ perceived oral health and frequency of dental visits. There is a statistically significant positive relationship between the mother’s brushing frequency and the mother’s brushing frequency of the young child. There is a statistically significant positive relationship between the mother’s assessment of oral health and the mother’s frequency of brushing the child’s teeth.
Riggs et al, 2016 Australia [109]	Qualitative research Multicentric 1 year	14 Afghan women 10 Sri Lankan women 3 Sri Lankan doctors 19 dental personnel 10 midwives	Describe the knowledge and beliefs of Afghan and Sri Lankan women regarding maternal oral health Describe barriers to accessing dental care during pregnancy Present the perspectives of maternity and dental professionals regarding dental care for pregnant women	Semi-structured interview guide	Participatory method Focus groups		×	×	×	The study found: a perception among women and men that dental care is not harmful during pregnancy; lack of awareness among midwives and community members of the potential impact of poor maternal oral health; a general lack of awareness and a “priority access” policy allowing pregnant women to receive free dental care.
Rocha et al, 2017 Brazil [112]	Longitudinal study Multicentric 4 years	73	Analyse the influence of oral health conditions and sociobehavioural characteristics of the pregnant women on the development of caries and that of their children, after 4 years of follow-up	Questionnaire designed by the authors	Mother: questionnaire waived during an interview Child: home visits (periodic) and oral examination	×	×		×	14 factors are listed as being related to barriers and facilitators to dental care during pregnancy: physiological conditions, low importance of oral health, stigma of dentistry, fear/anxiety about dental treatment, mobility and safety, financial barriers, employment, time constraints, social support, lack of information, barriers from health professionals, advice from family and friends, and beliefs and myths about the safety of dental treatment Myths and beliefs about oral health and dental treatment during pregnancy seem to be the most common barriers, both for pregnant women and for dentists or other health professionals.
Rothnie et al, 2012 New Zealand [114]	Cross-sectional study Nulticentric 1 month	104	Assess the knowledge of expectant mothers in Dunedin regarding the oral health care of their future children	Questionnaire designed by the author	Self-administered		×		×	Less than half of the participants felt they had enough information about their child’s oral health needs. A quarter thought that tooth brushing should not begin until the age of two. Most believed that their child should not be seen by a dental professional until 2 years of age. Children’s oral health knowledge was lower among first-time mothers, younger women, those from low socio-economic groups and those who were not from New Zealand (NZ).
Shamsi et al, 2013 Iran [118]	Cross-sectional study Multicentric Period not specified	340	Describe the status of dental caries in a sample of pregnant Iranian women and the factors on which action could be envisaged	Questionnaire designed by the author	Self-administered	×	×		×	Most (82%) agreed that women should have a dental examination during pregnancy, but only 46% actually did so. There were significant positive correlations between the age of the participants and the DMFT scores. Knowledge of brushing frequency, flossing, and duration of brushing were significantly associated with their practice. Those with less than a high school education had significantly less oral health knowledge than those with at least a college education 73% of the women had never received advice from their doctor or midwife on the value of visiting a dentist. 77% responded that they would be willing to attend a dental examination as part of their prenatal care. Pregnant women had little knowledge of oral health and its relationship to systemic diseases, such as diabetes, nor to pregnancy outcome.
Sun et al, 2014 China [127]	Cross-sectional study Multicentric 6 months	2259	Investigate the usual use of dental care	Questionnaire designed by the author	Self-reported		×		×	The percentage of follow-up dental appointments was significantly lower among pregnant women with the following characteristics: age 30 years or younger, annual household income less than $8,000, brushing once a day or less, never flossing or rinsing their mouths, not paying attention to pregnancy-related oral health knowledge, and being dissatisfied with their individual dental hygiene behaviour.
Thomas et al, 2008 Australia [129]	Cross-sectional study Unicentric 5 months	388	Assess women’s knowledge and experiences of dental health during pregnancy and examine oral health self-management practices of pregnant women.	Questionnaire was developed from 3 validated questionnaires (1. The National Dental Information Telephone Survey (NDTIS); 2. Impact Profile on Oral Health (IBHP); 3. Comparison of WHO Oral Health Care Systems) as well as additional questions designed by the authors.	Self-administered		×		×	There was a significant association between dental knowledge and practice, education, and socioeconomic status. Women with less education and lower socioeconomic status were more likely to have poor periodontal health than women with more education and a higher socioeconomic status.
Vann et al, 2010 USA [134]	Cross-sectional study Multicentric Period not specified	1158 mother/child pairs	Investigate caregivers’ oral health literacy, their oral health knowledge, behaviours, and oral health status of their preschool children	Data from child/caregiver dyads participating in the Carolina Oral Health Literacy (COHL) project REALD-30	Face-to-face interview		×	×	×	After adjusting for age, education, and number of children, a low literacy score (< 13 REALD-30) was associated with less knowledge and a poorer reported oral health status. Caregivers’ oral health literacy has a multidimensional impact on oral health status on infants and young children (including nighttime bottle feeding and lack of daily brushing/cleaning).
Vilella et al, 2016 Brazil [137]	Cross-sectional study Unicentric period not specified	175	Assess the oral health knowledge of pregnant women and its association with social determinants and knowledge of dietary and oral hygiene habits among children	REALD-30	Semi-structured questionnaire applied in the form of an interview, individual interviews		×	×	×	A positive correlation was found between literacy scores and knowledge, income, and the age at which infants first consumed sugar in their diet. The literacy scores were higher for pregnant women who had more than 8 years of education, who belonged to higher socio-economic classes, and who were employed. A significant correlation was found between the oral health literacy score and knowledge. Lower social determinants were associated with lower oral health literacy scores.
Wapniarska et al, 2016 Poland [141]	Cross-sectional study Multicentric 5 years	146	Assess parents’ knowledge of oral hygiene and dental caries prevention for infants and young children	Self-designed questionnaire consisting of 31 questions, 10 of which were analysed for this publication.	Self-administered				×	Young parents’ knowledge of prevention and children’s oral hygiene is still alarmingly low. Parents go to the dentist far too late, when caries is already active in their child’s mouth. Fathers’ knowledge of prevention and oral hygiene of children is much lower than that of mothers.
Wigen et al, 2014 Norway [143]	Retrospective study Multicentric 5 years	1348	Study how family characteristics and health behaviour during pregnancy and early childhood influence the development of caries in children of preschool age	Data from the MoBa study (Maternal and Child Cohort Study)	Data files	×	×		×	Toothbrushing frequency at 1.5 years of age was stable through preschool. Children whose teeth were brushed twice a day at 1.5 years of age brushed more often twice a day at 3 years of age and at 5 years of age than other children. The development of caries in preschool was related to the child’s and mother’s risk behaviour in early childhood and to the characteristics of the families at risk. Children with both parents of Western origin or with an older sibling are more likely to have more stable hygiene habits.
Wu et al, 2014 China [145]	Cross-sectional study Multicentric Period not specified	832	To examine the socioeconomic and behavioural risk factors for periodontal disease in women of childbearing age and evaluate the extent of public awareness of the association between oral health and pregnancy in China.	Questionnaire designed by the author	Self-administered		×		×	Periodontal health was not considered a priority. Abnormal body mass index was significantly associated with self-reported periodontal disease. Minimal mental stress, high annual household income, advanced oral hygiene aids, and knowledge of the link between pregnancy and periodontal disease were associated with a decreased incidence of reported periodontal disease. Participants who were aware that periodontal problems are harmful to infants and mothers better used oral hygiene aids and were statistically significantly less likely to report a periodontal symptom than other women.
Zhong et al, 2015 China [150]	Cross-sectional study Unicentric Period not specified	100	Assess pregnant women’s knowledge and beliefs about gingivitis in pregnancy and children’s oral health	Questionnaire designed by the author	Self-administered		×		×	Pregnant women generally lacked oral health knowledge and awareness of oral health care during the prenatal period.

DCC*: Dental check-up; SDD*: Socio-demographic data; OHL*: Oral Health Literacy; KAP*: Knowledge, Attitude, Practice

The included references were published between 2005 and 2022. The selected references show geographical and cultural diversity. The greatest proportion of studies (n = 14) were conducted in the United States,^11,51,17,18,21,24,36,48,55,82,84,87,106,135^ followed by nine in India,^9,41,46,58,68,94,99,100^ four in Brazil,^14,81,112,138^ four each in Australia,^43,64,110,130^ Iran,^10,52,85,118^ Poland,^23,42,65,142^ and China,^53,129,146,150^ three in Nigeria,^2,34,99^ two each in England,^27,54^ France,^30,102^ Nepal,^47,75^ and Spain,^73,80^ and one each in Canada,^8^ Greece,^33^ Jordan,^7^ Turkey,^98^ New Zealand,^114^ Norway,^144^ Oman,^13^ Pakistan,^67^ Palestine,^63^ Saudi Arabia,^40^ Singapore,^12^ Sudan,^56^ and Switzerland.^70^

All studies collected sociodemographic data from participants.

Of the 67 studies included, six were qualitative, one was a mixed method and 60 were quantitative, including 52 cross-sectional studies, three case-control studies, two retrospective studies, two prospective studies and one systematic review.

The quantitative research was based on questionnaires. Some of the questionnaires concerning health status were taken from validated questionnaires from large national health surveys (the National Health and Nutrition Examination Survey, the National Health Interview Survey, and the Maternal and Infant Health Assessment in the USA^24^) or from surveys specifically on oral health (Adult Dental Health Survey in England,^27^ the Fourth Oral Health Questionnaire in China^53^). In most of the studies, the questionnaires concerning the measurement of KAP in pregnant women were developed by the authors themselves and five studies used validated questionnaires. In the study by Thomas et al,^129^ the collection tool was developed from three validated questionnaires: the National Dental Telephone Interview Survey (NDTIS), the Oral Health Impact Profile (OHIP), and the World Health Organization’s Comparing Oral Health Care Systems. In the study by Hunter et al,^55^ the validated questionnaire used was the Oral Health Assessment Questionnaire (OHAQ). Concerning the measurement of health beliefs, Hosseintalei et al^52^ used a validated questionnaire derived from the Health Belief Model (HBM), and Balan et al^12^ used three questionnaires, including the validated Pregnancy Risk Assessment Monitoring System (PRAMS) questionnaire and two others (Rustvold Oral Health Knowledge Inventory [ROHKI] and Oral Health Attitudes Questionnaire [OHAQ]) based on the HBM and applied to oral health. Concerning the measurement of OHL, one study used the BRIEF Health Literacy screening tool^82^ and three studies used the validated REALD-30 Rapid Estimate of Adult Literacy in Dentistry (REALD-30) questionnaire.^51,134,137^ The BRIEF tool questionnaire is a short self-report instrument (three questions) developed to identify patients with inadequate health literacy. The REALD-30 questionnaire^31^ is based on the Rapid Estimate of Adult Literacy in Medicine (REALM), which is a 66-item medical recognition instrument. It includes 30 dental terms, with an emphasis on disease-specific terms, including aetiology, anatomy, prevention, and treatment. Of the three levels of OHL,^149^ this questionnaire assessed the functional level. One study used the Maternity Social Support Scale (MSSS) to assess social support during pregnancy, as social support can influence health behaviours.^82^

### Characteristics of Oral Health Knowledge, Attitudes, Practices, and Literacy

The oral health knowledge component was addressed in 52 articles on pregnant women and 17 on children. This component includes general knowledge related to oral health,^2,7,9,14,15,18,23,34,42,47,52,53,56,58,65,68,70,73,81,82,85,87,89,100,118,146^ knowledge of behavioural factors that influence oral health (hygiene and dietary habits),^2,9,15,23,27,34,42,43,53,56,65,102^ and the recognition of signs of oral pathology.^27,42,56^ More specifically, in relation to pregnancy, 31 articles focused on knowledge of the links between oral health and pregnancy,^7,8,9,11,12,14,30,36, 40,41,42,43,46,53,48,58,65,68,70,84,85,94,98,99,100,102,110,114,118,129,146^ six addressed knowledge of the relationship between maternal and child oral health,^34,40,43,81,110^ and ten addressed maternal knowledge of child oral health.^14,23,41,42,43,51,73,84,89,114^ The main associations shown were between knowledge and educational level or professional status and between knowledge and ethnic origin. Pregnant women with greater education or a higher socioeconomic status had statistically significantly higher oral health knowledge scores, higher awareness scores concerning the association between oral health and pregnancy, and oral treatment options during pregnancy.^2,9,12,15, 23,58,65,73,82,85,87^ Pregnant women in the low-income group had a statistically significantly higher experience of dental problems during pregnancy than those in the higher-income groups.^12,24,118^ The distribution of the knowledge component does not appear to be evenly distributed according to ethnicity. African American and Hispanic/Latino women reported statistically significantly lower mean knowledge scores on child oral health than did white women;^11^ there was a statistically significant difference between African American and Hispanic women concerning knowledge about the relationship between pregnancy and gingivitis.^18^

The ‘attitude’ component provides information on the importance the pregnant woman places on her oral health and that of the unborn child, her perceptions, and her beliefs. Attitude was examined in 26 articles^9,11,12,17,24,42,43,46,52,53,54,55,56, 58,63,64,65,70,80,85,98,100,102,118,129^ for pregnant women and in one article for the child.^141^ The concept of oral health beliefs was examined in 26 articles for pregnant women and 13 for the child. Beliefs identified included the importance of oral health in relation to general health,^11,17,34,68,89,133,141^ the impact of pregnancy on oral health, the issue of caring for primary teeth, and the inevitability of caries in children.^11,14,18,34,38,39,51,89^ Some of these beliefs contribute to the use of dental care during pregnancy. Women who had visited the dentist in the previous 12 months had higher belief scores about the importance of the child’s oral health.^11^ Several studies examined the relationship between oral health beliefs and ethnicity. Hispanic women were more likely than white or black women to believe that routine dental care was unsafe during pregnancy, and also more likely to believe that tooth loss during pregnancy was a normal phenomenon.^18^

The ‘practical’ component was the most studied (54 of the 67 included articles), and three main themes were listed: dental habits (oral hygiene behaviour and the use of preventive care), dietary habits (frequency of sugar intake), and the use of care during pregnancy. The question of addictions (alcohol and tobacco consumption during pregnancy) was raised in four articles.^48,80,112,129^ Concerning the child, the questions concerned the feeding of the newborn: breastfeeding, bottle feeding (frequency of intake, night-time bottle feeding, early tooth decay of the child), diet (frequency and type of consumption, juice, sweet snacks), dental hygiene habits (toothbrushing, frequency), sucking habits (pacifier, sweetened pacifier, thumb), and utilisation of dental services as well as follow-up of the child by a dentist. The practice component was studied mostly in relation to knowledge, maternal education, and ethnicity. Oral hygiene practices differed statistically significantly by ethnicity.^16,36,55^ Black women were more likely to report brushing only once a day or less and Hispanic women were more likely to floss daily.^17^

Statistically significant associations were found between the oral hygiene practices of pregnant woman (frequency and duration of brushing, use of dental floss, visits to the dentist, etc.) and the level of education,^56^ knowledge related to brushing techniques,^2,34,56,80,129^ and the oral health practices of the mother for the child.^144^

### KAP Components and Oral Health Status

In several studies, an oral examination was sometimes performed simultaneously with questionnaire data collection. A positive correlation was found between the self-assessed knowledge of pregnant women and their oral health; those with a good oral health status often rated their dental knowledge as sufficient or very good, whereas those with a poor oral health status more often rated their knowledge as limited or insufficient.^42,145^ A statistically significant inverse relationship was found between the mean knowledge score and the mean DMF (index counting the number of decayed, missing, or filled teeth due to dental caries)^52^ and between education and the DMF score.^63^ In addition, pregnant women who had some knowledge of the association between pregnancy and oral health were less likely to experience periodontal symptoms.^9,145^ Those with the lowest or intermediate level of education had a higher percentage of bleeding on probing, a higher plaque index, and a greater likelihood of untreated carious lesions.^24^

There was no difference in the prevalence of caries between the low- and high-income groups when patients were aware of reimbursement for care during pregnancy, whereas it was higher in the low-income group when patients were not aware of reimbursed care during pregnancy.^30^

Data from clinical examinations showed statistically significant associations between periodontal status (bleeding on probing, probing depth), caries risk, and hygiene practices of pregnant women.^8,9,12,24,56,64^ Women who brushed at least twice a day before pregnancy were less likely to have gingival bleeding^9,64^ and had fewer decayed teeth than other women.^56,80^

### KAP Components and Health Care Utilisation

Healthcare utilisation was examined in two ways: general use except pregnancy (family dentist, frequency and reason for use, history of need for dental care before pregnancy) in 23 articles,^2,8,9,11,13,17,34,36,40,43,54,56,64,67,80,85,87,98,106,110,112,114,146^ and the use of dental services during pregnancy in 19 papers.^2,8,36, 40,48,65,70,81,82,85,94,98,99,102,106,110,114,129^ Visits to a dental surgeon during pregnancy appear to be closely related to several factors, including pre-pregnancy healthcare use patterns, the weight of information provided by prenatal care professionals, family and socioeconomic background (including marital status, private insurance, and income level), prority level as well and convictions and beliefs.^8,15,17,43,48,58, 63,106,110,144^

The frequency of preventive visits (outside of pregnancy) and the time since the last dental visit were statistically significantly associated with dental service use during pregnancy.^8^ Mothers who reported regular dental visits (every 6-12 months when not pregnant) were approximately 10 times more likely to use dental care during pregnancy than mothers who reported a visit every two years.^48^ The frequency of visits was related to the KAP components.^8,27,48,129^ Mothers who consulted regularly had more knowledge about the methods of preventing caries in children (including the need for annual preventive consultations) and the importance of dental follow-up during pregnancy than those who consulted only occasionally.^30,34^ Mothers who had consulted the dentist during pregnancy had statistically significantly higher attitude scores on the importance of consulting a dentist for a health check-up, a preventive approach or in case of dental problems, and a statistically significantly higher perception of the importance of oral hygiene.^8^ Concerning oral health status, pregnant women who visited the dentist regularly before pregnancy (every 6 to 12 months) had fewer dental caries (p > 0.05), more restored teeth (p > 0.05), and more present teeth (p > 0.05).^80^ Not having a dental visit in the six months prior to pregnancy (compared to having had one one) was associated with bleeding on probing and a higher plaque index (p < 0.01-05) as well as more untreated caries (p < 0.001).^24^

Moreover, the use of healthcare is also dependent on the use of addictive substances: mothers who reported smoking before pregnancy or drinking alcohol during pregnancy were statistically significantly less likely to report a visit to the dentist during pregnancy.^48^

Twenty articles addressed barriers to seeking care during pregnancy. Intrinsic barriers included lack of knowledge, perceived stress of the impact of dental care during pregnancy, prioritisation, cultural aspects, and the ability to plan for oral health. Barriers extrinsic to the individual included a lack of information and referral, lack of access to dental offices, geography, and the time and cost of care.^8,13,17,43,47, 54,63,75,82,106,110,118^ Thus, most women reported that if they had been aware of the links between oral and general health, they would have paid more attention to maintaining good oral health.^46^ In the study by Shamsi et al,^118^ 73% of women had never been advised by their doctor or midwife of the importance of visiting a dentist during pregnancy, and 77% responded that they would have been willing to attend a dental examination as part of their antenatal care. Mothers reported that advice from their various healthcare practitioners about the lack of safety of dental care was a barrier to accessing dental care.^63,102^

In addition, for patients who received dental care during pregnancy, there was no statistically significant relationship between caries prevalence and poverty status, whereas it was statistically significant for patients who did not receive dental care during pregnancy.^30^ Mothers who perceived the costs and time constraints of dental care as important challenges had higher DMFT scores than other mothers.^63^

### KAP Components and Oral Health Literacy

Literacy as a determinant of oral health of pregnant women has been studied with the knowledge component alone^51^ or in combination with the knowledge and practice^23,82,110,137^ and behaviour^23,134^ components.

A positive correlation was found between higher knowledge levels and higher levels of OHL. Higher REALD-30 scores were associated with correct responses to two knowledge items: the use of fluoride as a preventive measure and the risk of spreading oral infections.^137^ A statistically significant positive correlation was also found between parents’ literacy level and their knowledge about children’s oral health.^82,134^ After adjusting for age, ethnicity, education and the number of children, low oral literacy scores (< 13 REALD-30) were associated with lower levels of knowledge and a low perception of oral health; higher literacy scores were associated with a better perception of self-reported oral health.

Pregnant women with low OHL had statistically significantly lower scores on the practical component concerning eating habits (diet and frequency of intake) and oral hygiene practices. A positive correlation was found between OHL level, knowledge score, and income (social determinants) and the age at which participants intended to introduce sugar into the infant’s diet.^137^

Concerning dental habits, lower literacy scores were found among parents who did not report daily toothbrushing than among those who did, and parents who put their child to bed with a bottle compared to those who never did so.^133^

## Discussion

In this scoping review, we identified 67 studies that investigated oral health knowledge, attitudes, practices, and literacy of pregnant women. Our findings indicate the limited amount of research specifically addressing the simultaneous study of the three KAP components and OHL of pregnant women (only one study),^134^ and the limited number of studies addressing the three KAP components (four studies).^13,46,58,85^ In almost all studies, the analysis focused solely on the relationship between the knowledge component and one of the factors: attitude, practice, or literacy. We also found references to the concept of health behaviour without further defining the link to KAP and literacy factors. Although the KAP and literacy components were only partially investigated in several studies, the results provide evidence of common characteristics and associations across many studies.

Knowledge is the principal component that was found in all studies. It was mostly studied from the perspective of the pregnant woman’s knowledge of oral health in general, oral health during pregnancy, and, more rarely, the oral health of the unborn child. The cognitive aspect is predominant, with a presupposition that it is enough to know about something in order to do it. The pregnant woman’s knowledge was generally evaluated based on her ability to define the concepts studied (for example, definition of caries or periodontal disease), state the different elements involved (hygiene, diet, use of dental services, prevention, etc), and identify misconceptions. The level of oral health knowledge of the pregnant woman appears to be linked to her living environment (place, sociocultural context, and value system of the group in which the pregnant woman was raised), level of education, and socioprofessional level, all of which influence her attitudes and practices. Although some women had a satisfactory level of general knowledge about oral health, it was not very specific concerning the oral health of the unborn child (in terms of caries risk factors, hygiene practices, follow-up, the importance of primary teeth, and the need to care for them) and was often accompanied by contradictory practices. These results raise questions concerning the understanding of the information transmitted and the mother’s ability to implement the recommendations.

Attitude was the least frequently studied component, and the research was mainly based on the perceptions, beliefs, and health representations (psychological dimension) of pregnant women, taking into account sociocultural specificities.^35^ Attitude is often considered a relatively fixed variable that serves as an intermediary between the woman’s knowledge, living situation, and practice of oral health and can be influenced by the weight of beliefs. In this scoping review, the complexity of the ‘attitude’ component as proposed in Quissel et al’s model^105^ is not apparent. In this model, oral health attitude is referenced within the expected mediators and includes the locus of control, the feeling of self-efficacy, and the perceived importance of the behaviour related to oral health, in relation to the Health Belief Model (perception of the severity of oral pathologies, expected benefits of having favourable behaviours in oral health, and perceived barriers).^105^ Rondier and Bandura^113^ defined the self-efficacy construct as the perception that an individual is able to perform a behaviour despite the presence of barriers and obstacles, provided that he/she has the minimum knowledge and considers it to be an important dimension of his/her life. This was not identified in these studies as a variable to be studied in the understanding of the oral health behaviour of pregnant women.

Finally, the practical component was the second-most studied component after knowledge. The actions studied were those that are observable and quantifiable, such as the modalities of dental-surgeon services (visit before and during pregnancy, participation in a prevention programme, follow-up of therapeutic planning, emergencies, etc), oral hygiene, and dietary habits. The oral health practices of women before pregnancy appear to be a predictor of the practices of the woman during pregnancy, whether it concerns dental habits, diet, or the use of oral health care.^3,60,136^ Thus, the use of oral healthcare and prevention during pregnancy are likely related to the frequency of use before pregnancy. It also appears that women’s practices in terms of utilisation of dental services are influenced, among other things, by the beliefs that oral pathologies are inevitable during pregnancy and are aggravated by the fear of seeking medical care during this period, accompanied by erroneous knowledge concerning the safety of dental care during pregnancy.

Furthermore, the results obtained clearly show the link between each of the KAP components and socioeconomic level. As in the general population, the oral health status of pregnant women is characterised by social inequalities in health. These results should be considered and associated with the known data on the presence of strong social inequalities in oral health from the earliest age. Thus, if the pathology is mainly concentrated in children from disadvantaged social backgrounds, it would be correlate with the oral health status of the pregnant woman. In other words, the child’s lifetime oral health may be linked to that of his/her parents according to the transgenerational inheritance described by Trannoy^130^ (whether in the transmission of health status, preventive behaviours, or professional status) and partly dependent on the characteristics of the mother during her pregnancy^121,122,130^ and her literacy level.^38^

Our study had several limitations. The results obtained on the influence of KAP and literacy components in the oral health behaviours of pregnant women do not allow us to create an exhaustive model of the links between all components, as only one study dealt with the subject as a whole. The question remains unanswered concerning the oral health skills that need to be acquired by pregnant women in terms of knowledge, skills, and attitudes, taking into account the level of literacy and socioeconomic factors. Although the interrelationships between KAP components are evident in these studies, the results do not provide a comprehensive understanding of the oral health behaviours of pregnant women.

Characterisation of the oral health KAP components of pregnant women is only a first step towards developing a consensus on a common set of educational needs and expected skills for good oral health and is not in itself sufficient. There are already examples of educational interventions in a number of antenatal care settings. Vamos et al,^131^ in his systematic review in 2015, and Riggs et al^109^ in a Cochrane meta-analysis on oral health interventions for pregnant women, discuss the fact that such actions are generally highly oriented towards the assessment of knowledge and concrete practices, but do not take into account the literacy level of the pregnant women or their ability to understand and use the information transmitted. Oral health interventions for pregnant women should be based on the reference models of knowledge transfer and evaluated by more systematic studies.^20,115,131^ Programmes structured around a planning framework known as Intervention Mapping propose an ecological model that considers individual and environmental determinants.^44^ Among such determinants, the identification of psychosocial factors and the assessment of skills (KAP and literacy) is an essential preliminary step in the development of a model that combines the processes of behaviour change and decision making while taking into account the concept of empowerment. It should be considered jointly with the aim of reducing social inequalities in health.

## Conclusion

This scoping review is a first step in investigating the knowledge, attitude, practice, and literacy components of oral health behaviours of pregnant women. Although the links between knowledge, attitude, practice, and literacy have been studied, their complexities were not fully addressed and the results lack sufficient evidence to draw conclusions on the competencies that should be identified for planning oral health educational interventions for pregnant women. There is still a gap between the data in the recommendations of learned societies for maintaining good oral health and the reality of oral health behaviours of pregnant women. Thus, studies still need to be carried out that account for the level of literacy of pregnant women, the components of knowledge, attitudes (including the feeling of self-efficacy), and practices in oral health, the socioeconomic context of their lives, and the existence of cultural barriers. The aim is to estimate the needs of pregnant women as closely as possible, to reduce social inequalities in oral health and make the implemented educational actions more effective.
